# Supplementary material for: Reconciling Mining with the Conservation of Cave Biodiversity: A Quantitative Baseline to Help Establish Conservation Priorities
Source: PLoS One. 2016 Dec 20;11(12):e0168348. doi: 10.1371/journal.pone.0168348 (PMC5173368; doi:10.1371/journal.pone.0168348)
Supplement: S1 Dataset — (ZIP) [file pone.0168348.s002.zip › Taxa/Serra Norte/SN_2013/N4E/Lista N4E-07.pdf]

CAVIDADE N4E-0007

Classe	Ordem	Fam/Outros	Gên/Outros	Espécie	S-U	U-S	Zon.
Arachnida	Acari	(Parasitengonina)		sp.9		X	P
Arachnida	Acari	Trombiculidae		sp.	X		
Arachnida	Amblypygi	Phrynidae	*Heterophrynus*	*longicornis*	X		
Arachnida	Araneae	Drymusidae	*Drymusa*	*spelunca*	X	X	E/P
Arachnida	Araneae	Ochyroceratidae	*Ochyrocera*	sp.3	X	X	E
Arachnida	Araneae	Ochyroceratidae	*Speocera*	sp.1	X	X	P
Arachnida	Araneae	Pholcidae	*Mesabolivar*	sp.1	X	X	P
Arachnida	Araneae	Symphytognathidae	*Anapistula*	sp.1		X	P
Arachnida	Araneae	Theridiosomatidae	*Plato*	sp.1	X	X	E
Arachnida	Opiliones	Sclerosomatidae	Gagrellinae	sp.	X		
Arachnida	Palpigradi	Eukoeneniidae	*Allokoenenia*	sp.1		X	E
Arachnida	Pseudoscorpiones	Chthoniidae		sp.	X		
Chilopoda	Scutigeromorpha	Pselliodidae	*Sphendononema*	*guildingii*	X	1	P
Diplopoda	Polydesmida	Pyrgodesmidae		sp.1		X	E
Diplopoda	Polydesmida			jovem	X		
Entognatha	Collembola	Isotomidae		sp.1		X	E/P
Entognatha	Collembola	Sminthuroidea		sp.2		X	P
Entognatha	Collembola			sp.	X		
Entognatha	Diplura	Campodeidae		sp.1	X	X	P
Entognatha	Diplura	Japygidae		sp.2		X	E
Insecta	Coleoptera	Carabidae	*Gen*.n.	sp.17		X	E
Insecta	Diptera	Culicidae		sp.	X		
Insecta	Diptera	Psychodidae	Phlebotominae	sp.		X	P
Insecta	Diptera	Psychodidae	*Pintomyia*	*gruta*	X		
Insecta	Diptera	Psychodidae	*Sciopemyia*	*sordellii*	X		
Insecta	Diptera	Tipulidae	Tipulinae	sp.		X	P
Insecta	Homoptera	Cixiidae		jovem	X	X	P
Insecta	Hymenoptera	Formicidae	*Brachymyrmex*	sp.2		X	E
Insecta	Hymenoptera	Formicidae	*Camponotus*	sp.1	X	X	P
Insecta	Hymenoptera	Formicidae	*Crematogaster*	sp.1		X	P
Insecta	Hymenoptera	Formicidae	*Dolichoderus*	aff. *bispinosus*		X	P
Insecta	Hymenoptera	Formicidae	*Paratrechina*	sp.	X		
Insecta	Lepidoptera	Tineoidea		sp.	X		
Insecta	Orthoptera	Phalangopsidae	*Paraclodes*	sp.	X		
Insecta	Orthoptera	Phalangopsidae	*Phalangopsis*	sp.	X		
Insecta	Psocoptera			jovem	X	X	E
Gastropoda	Pulmonata	Subulinidae	*Lamellaxis*	sp.	X		
Gastropoda	Pulmonata	Subulinidae		sp.3		X	P
Gastropoda	Pulmonata	Systrophiidae	*Happia*	sp.	X		
Malacostraca	Isopoda	Scleropactidae		sp.	X		
Malacostraca	Isopoda	Scleropactidae	*Circoniscus*	*buckupi*	X		
Amphibia	Anura			sp.		1	P
Mammalia	Chiroptera	Emballonuridae	*Peropteryx*	sp.	3	1	P
